# New native *Bacillus thuringiensis* strains induce high insecticidal action against *Culex pipiens pallens* larvae and adults

**DOI:** 10.1186/s12866-023-02842-9

**Published:** 2023-04-13

**Authors:** Xinmin Ma, Jianjian Hu, Chengsong Ding, Roxana Portieles, Hongli Xu, Jingyao Gao, Lihua Du, Xiangyou Gao, Qiulin Yue, Lin Zhao, Orlando Borrás-Hidalgo

**Affiliations:** 1 Joint R and D Center of Biotechnology, RETDA, Yotabio-Engineering Co., Ltd, 99 Shenzhen Road, Rizhao, 276826 Shandong People’s Republic of China; 2 State Key Laboratory of Biobased Material and Green Papermaking, Shandong Provincial Key Lab of Microbial Engineering, Qilu University of Technology (Shandong Academic of Science), Jinan, People’s Republic of China

**Keywords:** *Bacillus thuringiensis*, Crystals, Endotoxins, Profiling, *Cry* and *cyt* genes

## Abstract

Mosquitoes of many species are key disease vectors, killing millions of people each year. *Bacillus thuringiensis*-based insecticide formulations are largely recognized as among the most effective, ecologically safe, and long-lasting methods of managing insect pests. New *B. thuringiensis* strains with high mosquito control effectiveness were isolated, identified, genetically defined, and physiologically characterized. Eight *B. thuringiensis* strains were identified and shown to carry endotoxin-producing genes. Using a scanning electron microscope, results revealed typical crystal forms of various shapes in *B. thuringiensis* strains. Fourteen *cry* and *cyt* genes were found in the strains examined. Although the genome of the *B. thuringiensis* A4 strain had twelve *cry* and *cyt* genes, not all of them were expressed, and only a few protein profiles were observed. The larvicidal activity of the eight *B. thuringiensis* strains was found to be positive (LC50: 1.4–28.5 g/ml and LC95: 15.3-130.3 g/ml). Bioassays in a laboratory environment demonstrated that preparations containing *B. thuringiensis* spores and crystals were particularly active to mosquito larvae and adults. These new findings show that the novel preparation containing *B. thuringiensis* A4 spores and crystals mixture might be used to control larval and adult mosquitoes in a sustainable and ecologically friendly manner.

**Supplementary Information:**

The online version contains supplementary material available at 10.1186/s12866-023-02842-9.

## Introduction

Mosquitoes are recognized as one of the most important groups of insects in transmitting many major infectious illnesses, such as malaria, filariasis, dengue, Rift Valley and yellow fevers, chikungunya and Zika fevers, and Japanese encephalitis, which cause millions of deaths each year [1, 2]. The mosquito is anthropophilic and domestic, with daily hematophagic activity, and its adaptive success is related to favorable domestic or peridomestic environments, including water breeding places, fast growth, and desiccation-resistant eggs [3]. These features lead to obstacles in managing this insect and, as a result, to a global public health problem.

Furthermore, the widespread and intense use of chemical pesticides for mosquito control has resulted in a number of environmental and human health issues, including the disruption of natural-biological control mechanisms, the development of mosquito resistance, and unfavorable impacts on beneficial species [4, 5]. Specifically, the mosquito species *Culex pipiens pallens* has had a high incidence in Shandong province and constitutes a potential vector of different diseases including the West Nile virus in China. Unfortunately, this species has developed insecticide resistance debilitating effective control [6].

The formulation based on *B. thuringiensis* spore and crystal mixture is among the few alternatives to conventional chemical compounds for vector control. This has been isolated from soil, aquatic habitats like sewage, dead insects, herbivore dung, stored grains, phylloplane, and woods [7]. Because of its high specificity, safety, and efficacy in the control of a wide range of human disease vectors and agricultural pests, this facultative aerobic, Gram-positive, spore-forming saprophyte soil bacterium has been used successfully as a biological insecticide for over six decades and accounts for 95% of all commercial bioinsecticides [7]. This bacterium is recognized as the most successful bioinsecticidal alternative available, with a toxicity toward a broad range of insect species such as Dipteran, Lepidopteran, and Coleopteran [8, 9].

During the sporulation stage, *B. thuringiensis* usually produces insecticidal proteins and δ-endotoxins [10, 11]. Hofte and Whitely were the first to describe four *cry* and two *cyt* δ-endotoxin genes with insecticidal activity [12]. The genetic diversity and toxic potential of *B. thuringiensis* strains can vary between countries and regions. For that reason, many *B. thuringiensis* strains were isolated and characterized around the world with the main objective of finding novel active *cry* genes to control resistant insects. The parasporal crystal form is an indication of Cry proteins content; hence, it is used as the first criterion of the classification of *B. thuringiensis* isolates [13–15]. The presence of *cry* and *cyt* genes can predict the insecticidal activity of a novel isolate. Due to the complexity of the δ-endotoxins action, it is also necessary to assess the frequency of the genes involved in larval mortality to understand the mode of action of these proteins in larval intestinal cells [16–18]. The combination of *cry* and *cyt* genes allows for high toxic activity, causing larval death within 24 h after exposure [19].

Additionally, there are many *B. thuringiensis* genes encoding proteins with demonstrated anti-dipteran activity such as *cry1, cry2, cry4, cry10, cry11, cry19, cry20, cry24, cry27, cry30, cry39, cry44, cry47, cry50, cry54, cry56, mpp60, tpp80, cyt1*, and *cyt2* genes [20]. Herewith, synergist combinations of Cry4Aa + Cry4Ba, Cry11Aa + Cry4Aa, and Cry11Aa + Cry4Aa + Cry4Ba were highly effective against *Aedes*, *Anopheles*, and *Culex*, respectively [21–26].

On the other hand, the initiation of larval toxicity occurs soon after the bacterial crystals are ingested, which are later solubilized in the alkaline pH of the larval midgut, releasing the protoxins. Different proteases are involved in *B. thuringiensis* protoxin activation of these protoxins into toxic forms, and the toxins bind to receptors on the intestinal epithelium in this active state in different groups (orders) of insects [27–30]. This interaction allows the creation of holes in the cell membrane, resulting in an ion imbalance that causes cell rupture and disintegration, eventually leading to the death of the insect [31].

*B. thuringiensis*-based products are extensively utilized, and several formulations have been designed. Suspension concentrates, wettable powders, and grained formulations are the most common commercial products [32]. Formulated bacteria are sprayed or dispersed across the surface of static or slow-moving water to suppress mosquito larvae. In addition, certain *B. thuringiensis* formulations using ice granules have been utilized [33]. A dispersible granule from *B. thuringiensis*, manufactured by Valent BioSciences Corporation was evaluated on field condition in Cabo Verde, with a mortality rate ranging from 43.1 to 90.9% [34]. Recently, a novel formulation known as “MosChito” rafts based on a low release delivery mechanism was recently created. This proved particularly successful in a variety of settings as an environmentally friendly solution [35].

Besides, evidence about the biological activity on mosquito adults was initially reported by Klowden et al. [36], who introduced *B. thuringiensis* subsp. *israelensis* crystals through enemas into the midguts of *Aedes aegypti* adult. The susceptibility from different mosquito species was evaluated after oral administration of solubilized *B. thuringiensis* [37]. A high mortality was observed when the *B. thuringiensis* was supplied in a saline and sucrose solution. Also, the ingestion of *B. thuringiensis* or Cry toxins in an attractive toxic sugar baits had a high mortality in different mosquito adults [38–40].

Until now, various alternatives have been used to controlling mosquitoes [41, 42]. However, although important progress has been made, it is still necessary to develop more efficient, sustainable, and environmentally friendly strategies. The existing biodiversity on the planet offers the possibility of finding alternatives for development and use in the solution of this important problem, or at least significantly reducing the negative effect of transmission by these vectors of serious diseases. *B. thuringiensis* has been isolated and investigated in a variety of ecologies since its discovery by Ishiwata in 1902. However, insecticides based on *B. thuringiensis* have not completely replaced dangerous chemical pesticides, and many ecologies remain unknown [43]. Therefore, this work is focused on the identification and characterization of *B. thuringiensis* isolates with naturally enhanced toxicity for biocontrol of mosquito larvae and adults.

## Materials and methods

### Isolation, identification, and characterization of *B. thuringiensis* isolates

Soil samples were collected in sterile plastic tubes from three different districts in Rizhao, Shandong, China, People’s Republic of China (Supplementary Fig. 1). The collected samples were transported to the laboratory and stored at 4 °C until processing for *B. thuringiensis* isolation. One gram of each soil sample was suspended in 10 ml of Luria-Bertani (LB) broth medium (yeast extract, 5 g/L: peptone, 10 g/L; sodium chloride, 5 g/L; pH 7). The mixture was then incubated in a shaker incubator at 30 °C for 4 h. After incubation, the samples were heated at 80 °C for 15 min. From each of these samples, 100 µl was spread on nutrient agar medium (yeast extract (3 g/L), peptone (5 g/L), sodium chloride (5 g/L), and agar (15 g/L)) (Sangon-Biotech, Shanghai) plates and incubated for 72 h at 30 °C. After that, each colony was grown on nutrient medium (yeast extract (3 g/L), peptone (5 g/L), and sodium chloride (5 g/L)) (Sangon-Biotech) plates supplemented with 100 µg/ml of penicillin G (Sangon-Biotech, Shanghai) and incubated at 30 °C for 96 h. *B. thuringiensis*-like colonies, white, large, and nearly circular with fine irregular margins and glossy, less glossy, or rough were selected.

A colony taken from a 24-hour culture of each *B. thuringiensis* isolate (A2, A4, A8, A10, B6, C2, C4 and C5) was suspended in 100 µl sterile distilled water in an Eppendorf tube and boiled in a water bath for 10 min. *B. thuringiensis* suspensions were then immediately cool-shocked at -20°C. This heat-shock process was repeated three times to allow complete cell lysis before centrifugation at 3,500× g for 10 min at 4°C. The resultant supernatant containing the crude DNA was used for PCR amplification [44]. The *16S rRNA* gene was used for molecular identification. A fragment of the *16S rRNA* gene (1.4 kb) was amplified by polymerase chain reaction (PCR) using the forward primer 27F 5’-AGAGTTTGATCCTGGCTCAG-3’, reverse primer 1492R 5’-GGTTACCTTGTTACGACTT-3’, and the SanTaq Plus PCR Master Mix (Sangon Biotech, Shanghai). The amplification process was conducted in a Thermal Cycler T100 machine (Bio-Rad Life Science Research, Shanghai, People’s Republic of China) using a Taq PCR Master Mix Kit (Qiagen, Hilden, Germany). The PCR reaction was as follows: 95^o^C for 10 min; 35 cycles of 95^o^C for 30 s, 55^o^C for 1 min, and 72^o^C for 1.5 min; and a final extension at 72^o^C for 10 min. The purified PCR fragment was sequenced using an ABI 3730 DNA sequencer (Applied Biosystems, Thermo Fisher Scientific, CA, USA). The *16 S rRNA* gene fragment sequence (1,147 bp) was identified using BLASTN homology searches [45] from the National Center for Biotechnology Information (NCBI) GenBank and the European Molecular Biology Laboratory-European Bioinformatics Institute (EMBL-EBI). Moreover, the *B. thuringiensis* strains identified were examined for the presence of parasporal inclusion bodies by light microscope. The bacteria were prepared as films on slides and heat fixed, then stained with Coomassie Brilliant Blue. Slides were observed with bright-field microscopy using a 100× oil immersion objective. Subsequently, crystals and spores morphologies were analyzed with Scanning Electron Microscopy (SEM) (SUPRA 55, Zeiss, Germany). A mixture of spores and crystals in sterile distilled was placed on SEM sample holder. Samples were dried at 37^o^C in oven, metalized and placed in vacuum chamber of SEM to capture images.

### PCR detection of *cry* and *cyt* genes contents

The supernatant containing the crude DNA from *B. thuringiensis* strains (A2, A4, A8, A10, B6, C2, C4 and C5) obtained according to the method mentioned above was used for PCR amplification [44], using the Hot Start Taq DNA polymerase and Master Mix Kit (QIAGEN Technologies, Germany). The PCRs were performed using the following reagents: 2.5 µL of GoTaq Flexi DNA polymerase buffer, 2.5 µL of dNTPs, 0.5 µL of MgCl2, 0.5 µL of each primer, 0.1 µL of GoTaq DNA polymerase (Promega) at approximately 50 ng/µL, and enough water (Milli-Q, Millipore) to reach a final volume of 12.5 µL. Amplifications were conducted in a Thermal Cycler T100 machine (Bio-Rad Life Science Research, Shanghai, People’s Republic of China) with the following program: an initial denaturation step at 94 °C for 5 min, followed by 35 denaturation cycles at 94 °C for 1 min, annealing (Table 1), extension at 72 °C for 1 min, and a final extension step at 72 °C for 7 min. The amplified products were stained with a mix of 2X Blue/Orange Loading Dye (Promega), loading buffer, and 1x GelRed Nucleic Acid Gel Stain (Biotium, Shanghai) dye and were separated on 1% agarose gels by electrophoresis. The sizes of the generated fragments were compared to the DNA Ladder 1 Kb molecular weight marker (Promega, Germany).

**Table 1:** Lists of primers used for amplification of *cry* and *cyt* genes from *B. thuringiensis*

Genes	Sequence 5’-3’	Product size	TM (°C)	Reference
***Cry1***	CTGGATTTACAGGTGGGGATAT TGAGTCGCTTCGCATATTTGACT	594	52	[44]
***Cry2***	GAGTTTAATCGACAAGTAGATAATTT GGAAAAGAGAATATAAAAATGGCCAG	500	50	[69]
***Cry4***	GCATATGATGTAGCGAAACAAGCC GCGTGACATACCCATTTCCAGGTCC	439	58	[55]
***Cry4Aa***	GGGTATGGCACTCAACCCCACTT GCGTGACATACCCATTTCCAGGTCC	777	50	[79]
***Cry4Ba***	GAGAACACACCTAATCAACCAAT GCGTGACATACCCATTTCCAGGTCC	347	52	[79]
***Cry4A***	TCAAAGATCATTTCAAAATTACATG CGGCTTGATCTATGTCATAATCTGT	459	50	[55]
***Cry4B***	CGTTTTCAAGACCTAATAATATAATACC CGGCTTGATCTATGTCATAATCTGT	321	50	[55]
***Cry10***	TCAATGCTCCATCCAATG CTTGTATAGGCCTTCCTCCG	348	51	[55]
***Cry10Aa***	ATTGTTGGAGTTAGTGCAGG AATACTTTGGATGTGTCTTGAG	995	50	[79]
***Cry11***	CGCTTACAGGATGGATAGG GCTGAAACGGCACGAATATAATA	342	50	[69]
***Cry11Aa***	CCGAACCTACTATTGCGCCA CTCCCTGCTAGGATTCCGTC	470	50	[79]
***Cry11Ba***	TACAGGATGGATAGGGAATGG TAATACTGCCATCTGTTGCTTG	608	52	[79]
***Cry24***	TTATCAATGTTAAGGGATGC ACTGGATCTGTGTATATTTTCCTAG	304	52	[69]
***Cry32***	TGGTCGGGAGAGAATGGATGGA ATGTTTGCGACACCATTTTC	676	48	[69]
***Cry44Aa***	CATTACACGGGGTGCGTTAT CCGCACTTACATGTGTCCAA	444	60	[80]
***Cyt1***	CCTCAATCAACAGCAAGGGTTATT TGCAAACAGGACATTGTATGTGTAATT	477	52	[69]
***Cyt2***	ATTACAAATTGCAAATGGTATTCC TTTCAACATCCACAGTAATTTCAAATGC	356	56	[69]
***Cyt1Aa***	AACTCAAACGAATAACCAAG TGTTCCTTTACTGCTGATAC	300	53	[79]
***Cyt1Ba***	AAGCAAGGGTTATTACATTACG CCAATACTAAGATCAGAGGG	698	54	[79]
***Cyt2Aa***	GCATTAGGAAGACCATTTG AAGGCTAAGAGTTGATATCG	361	53	[79]

### SDS-PAGE analysis of Cry and Cyt proteins

The *B. thuringiensis* (A4) culture with spore and crystal mixture was centrifuged, and the pellet was washed thrice with 1 M NaCl. The pellet was then washed 3x with distilled water. The spore-crystal pellet was resuspended in 50 mM NaOH and incubated at room temperature for 1 h to solubilize the crystal proteins. The purified crystal proteins were combined with 2x boiling buffer (containing 0.1% ß-mercaptoethanol, 1% SDS, 0.025% bromophenol blue, and 10% glycerol) at the ratio of 2:1, respectively. The samples were then boiled for 5 min along with a broad-range protein marker. The samples were loaded onto an SDS-PAGE gel with a 10% separating gel and 3% stacking gel. The electrophoresis was run at 100 V for 2 h. The gels were stained with a staining solution containing 0.025% Coomassie Brilliant Blue R250. Destaining was performed overnight with a solution containing ethanol, glacial acetic acid, and water at the ratio of 5:7:88, respectively. Additionally, the Bradford protein assay was used to measure the concentration of total protein [46]. Five dilutions of the protein standard (BSA) were prepared with a range of 5 to 100 µg protein. Protein solutions were assayed in triplicate. Bradford reagent (1.5 ml) was added to each tube and mix well. The tubes were incubated at room temperature for 5 min. Finally, the absorbance was measure at 595 nm.

### Preparation of the spore-crystal mixture for the bioassays

A volume of 40 µl of *B. thuringiensis* culture (A2, A4, A8, A10, B6, C2, C4 and C5 strains) preserved in glycerol was added to 40 ml of the LB medium in an Erlenmeyer flask. The *B. thuringiensis* fermentation was conducted in an incubator shaker at 37 °C and 120 rpm for 48 h. Subsequently, the culture was transferred to a fermenter with 40 L of medium for *B. thuringiensis* fermentation. The medium composition was the following: glucose, 10 g/L; corn powder, 10 g/L; soybean powder, 5 g/L; yeast extract, 1.5 g/L; peptone, 0.5 g/L; KH_2_PO_4_, 2.5 g/L; CaCO_3_, 0.5 g/L; and MgSO_4_•7H_2_O, 0.25 g/L; at a pH of 7.5 [47]. Batch fermentation (BAILUN BIO, Shanghai) was carried out under aseptic conditions during the whole run. The fermentation process was conducted at 37 °C and 300 rpm of agitation for 48 h (Supplementary Fig. 2). The pH was controlled automatically at 7.0. The airflow rate was maintained at 0.5–1.5 vol/vol. min (agitated at 300 rpm) through a sterile disk-type air filter (Millipore; diameter: 45 mm, pore size: 0.2 μm). Once complete, the fermentation was dried in a Speed Spray Dryer (Qiaofeng, Shanghai) with a frequency of 35 Hz and an inlet and outlet temperature of 150 and 80 °C, respectively. The drying process to obtain the powder was performed for two hours. Once the powder was dried and obtained, the final product was formulated by adding maltodextrin at a concentration of 10%, and it was vacuum packed (Supplementary Fig. 2). Around 100 g of orange and banana peels were crushed using a blender. An ethanol (95%) and extract mixture (5:1) was stored in a flask sealed for 72 h at room temperature. The mixture was filtered, and the extract was obtained by roto-evaporation (Yarong, Shanghai) at 60 °C. The 10% of extracted odorant oil was obtained and used during the experiments (Supplementary Fig. 2).

### Mosquito Bioassay using *B. thuringiensis* spores-crystals mixture

Larvae assay: The fermentations of A2, A4, A8, A10, B6, C2, C4 and C5 strains were used in the experiment. The bioassays were developed according to the guidelines for the laboratory testing of mosquito larvicide from the World Health Organization [48] with modifications. The *Culex pipiens pallens* species was used in the experiments due the incidence and prevalence in the Shandong province [6]. For each test, twenty-five third-instar larvae were transferred into a 50 ml glass Erlenmeyer flask containing 10 ml of chlorine-free tap water. Herewith, the *B. thuringiensis* powder with a concentration of 7.5 × 10^13^ colony forming units (CFU) per gram and 30 mg/g of total proteins was used in the experiments. The CFU was determined as follows: the *B. thuringiensis* dry powder (0.1 g) was dissolved in 10 ml of sterile water. The solution was diluted tenfold gradient and place on the nutrient agar medium. Three plates were used for each dilution. The plates were incubated overnight at 37 ℃. Finally, the colonies were counted in each plate (the effective range of colonies was between 30 and 300). The average value was multiplied for the dilution and calculate the concentration of the original dry powder solution. On the other hand, the different concentrations were prepared by serial dilution for each isolate. The control was prepared following the same procedure but without *B. thuringiensis*. The experiment was conducted in five repetitions. The bioassays were evaluated at 24 h after the application of *B. thuringiensis*, and the live and dead larvae were counted. The LC_50_ and LC_95_ were determined by a Probit analysis run in the POLO PLUS [49] program, based on the data on larvae mortality [50, 51] (Supplementary Fig. 3A).

Adult assay: Different treatments were used in the experiments with the following composition:

- Treatment A: mixture of water + *B. thuringiensis* A4 spore-crystal mixture (15.3 µg/ml and 30.6 µg/ml of total proteins) + odorant solution (10% v:v).
- Treatment B: mixture of water + *B. thuringiensis* A4 spore-crystal mixture (15.3 µg/ml and 30.6 µg/ml of total proteins).
- Treatment C: mixture of water + odorant solution (10% v:v).
- Treatment D: water.

A total of 85 adult mosquitoes of the same age were used in each treatment. The mosquito diet (10 ml) was replaced without spore-crystal mixture every day. The experiments were developed at 37 °C and 70% relative humidity. The bioassays were monitored every 24 h for two week, and the live and dead adult mosquitoes were counted. (Supplementary Fig. 3B).

### Mosquito maintenance

The mosquitoes were maintained under standardized conditions in cages with few modifications [52–54]. The adult male and female mosquitoes were fed on a diet of pig blood and sucrose 10% using an artificial feeder with a parafilm membrane at 37 °C and 70% relative humidity. This diet was replaced every day. Eggs were laid 2 days after blood-feeding and hatched within 2 days. About 200 hatched first larval instars were distributed into 1 L of deionized water in plastic trays (34 × 24 cm each). The pupae were separated daily, placed in polystyrene pots containing distilled water, and kept in their rearing cages until adult emergence.

### Data analysis

All the assays were performed three times with five or ten replicates of each group, and the values presented in the graphs/tables are means ± standard errors of the means. Statistically significant differences among the mean values were determined using a t-test and/or ANOVA at P < 0.05. P values of < 0.05 were considered statistically significant. The data were analyzed and processed using GraphPad Prism software (La Jolla, CA, USA).

## Results

### Isolation and identification of *B. thuringiensis* isolates

**Fig. 1:**
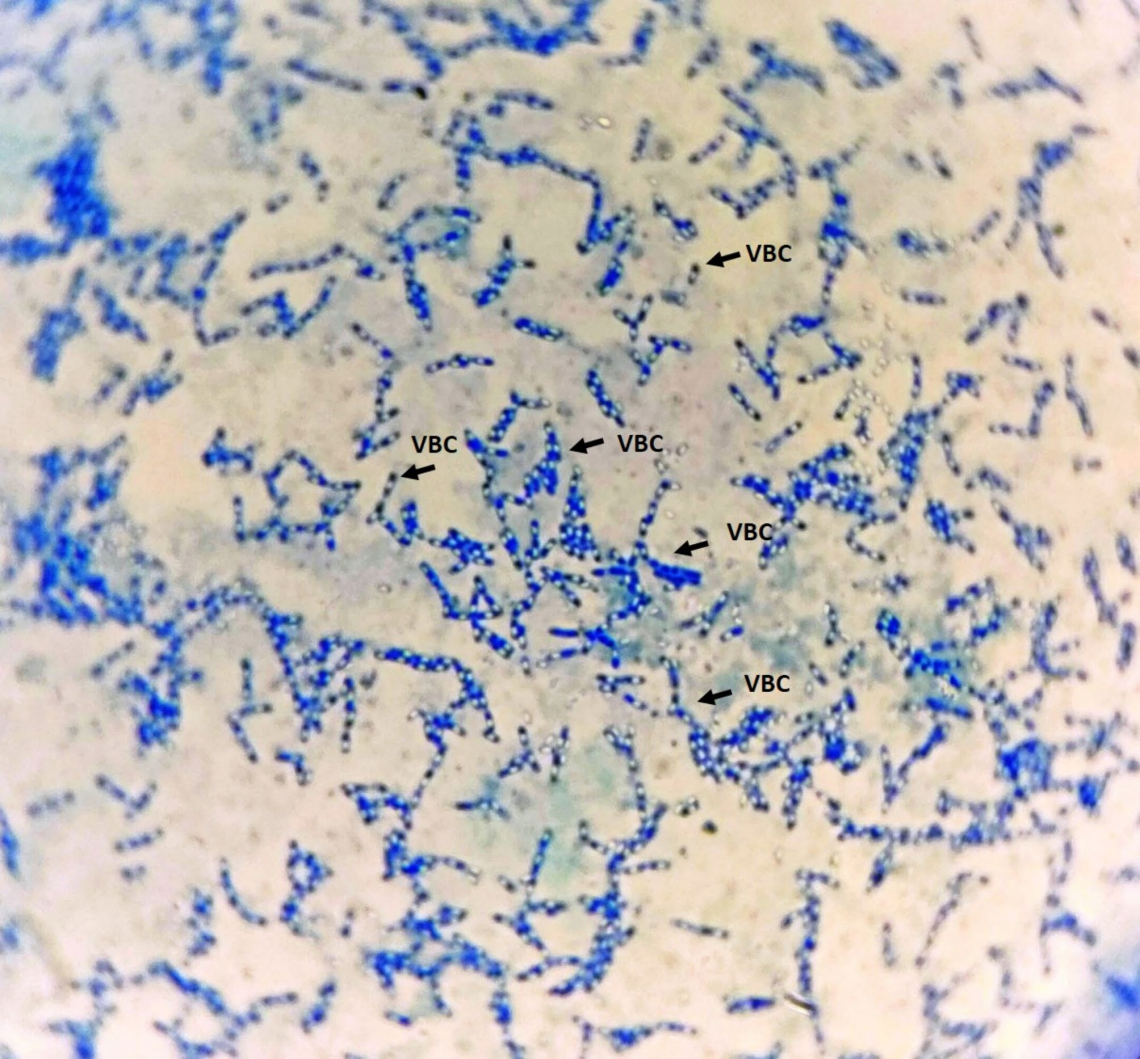
Light microscope photomicrographs of sporulated cultures of local A4 strain of *B. thuringiensis* (bar = 100 μm). VBC: Vegetative bacterial cell with spore and crystal inside

In total, 141 colonies of bacteria were isolated from different samples in the three analyzed districts of Rizhao City. Of this group of isolated colonies, only eight colonies (A2, A4, A8, A10, B6, C2, C4 and C5) corresponded to *B. thuringiensis*. The proportion of colonies of *B. thuringiensis* was low at 5.7% of the total (Table 2). All eight colonies of *B. thuringiensis* showed resistance to penicillin G. The structures of the eight isolates of *Bacillus thuringiensis* were studied under a light microscope. The microscopy allowed the identification of eight native potential *B. thuringiensis* isolates with characteristic *B. thuringiensis*-like colonial morphology and the presence of vegetative bacterial cell with spore and crystal inside (Fig. 1). The colony growth of these strains showed a white color, a large size, a nearly circular shape, fine irregular margins, and was usually glossy.

**Table 2:** Distribution of *B. thuringiensis* isolates in tested soils samples from different locations in Rizhao

Samples		Districts	Number of bacterial colonies	Number of *B. thuringiensis* colonies
**A**		Wulian	33	4 (A2, A4, A8, A10)
**B**		Lanshan	45	1 (B6)
**C**		Donggang	63	3 (C2, C4, C5)

**Fig. 2:**
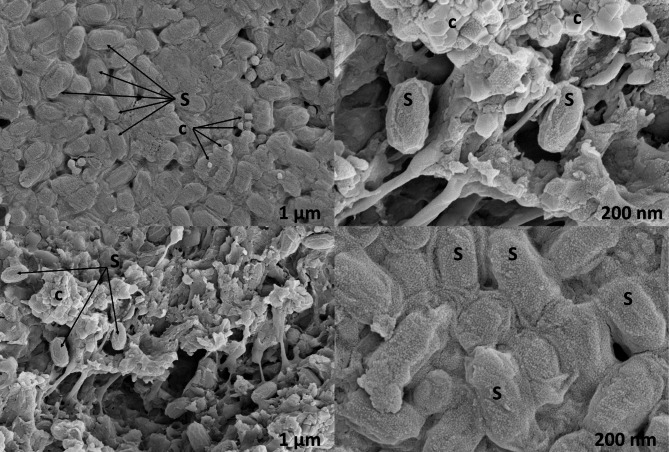
Scanning electron micrograph of spore and crystal proteins from *B. thuringiensis* native strain-A4. Spores (S) and crystal proteins (c) from *B. thuringiensis* native strain

Additionally, scanning electron micrographs allowed the identification of the typical bipyramidal and cuboidal crystals of *B. thuringiensis*. Smooth spherical shape, spherical shape with an undulated surface, spherical but deflated balloon shape, and spherical with one concave side and a pointy-edged shape were identified (Fig. 2). The total DNA of eight isolates (A2, A4, A8, A10, B6, C2, C4 and C5) was used for the amplification of rRNA fragment (1.4 kb) to finally confirm the identity of the isolates. According to the BLAST search in international databases, the eight isolates had high homology (99.50% of identity) with *B. thuringiensis* species.

### Molecular characterization of *B. thuringiensis* isolates

**Fig. 3:**
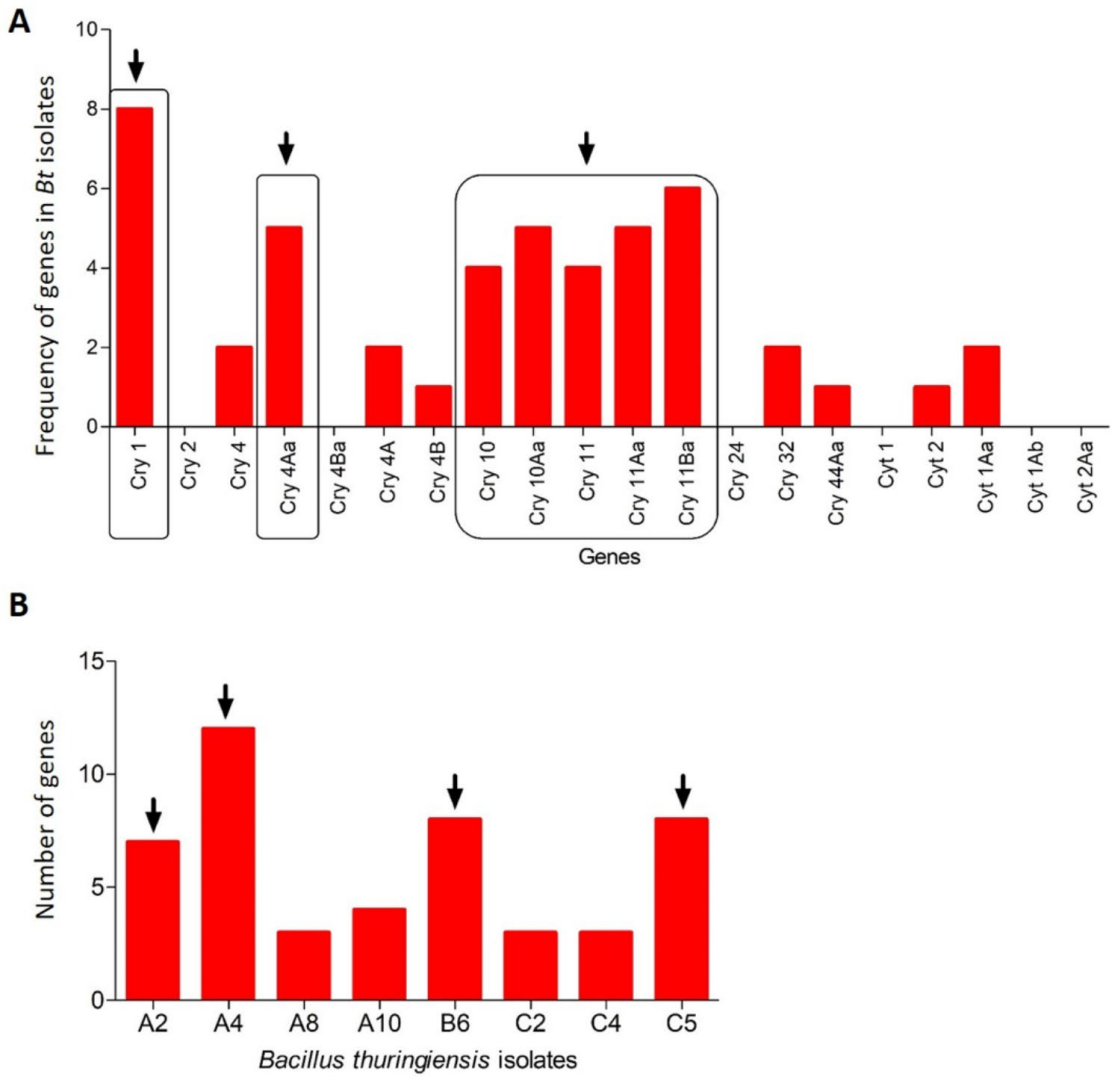
Frequency (**A**) and number (**B**) of the *cry* and *cyt* genes present in the new strains of *B. thuringiensis* from Rizhao. Arrows indicate the genes with more frequency and number of genes per *B. thuringiensis* isolates

**Fig. 4:**
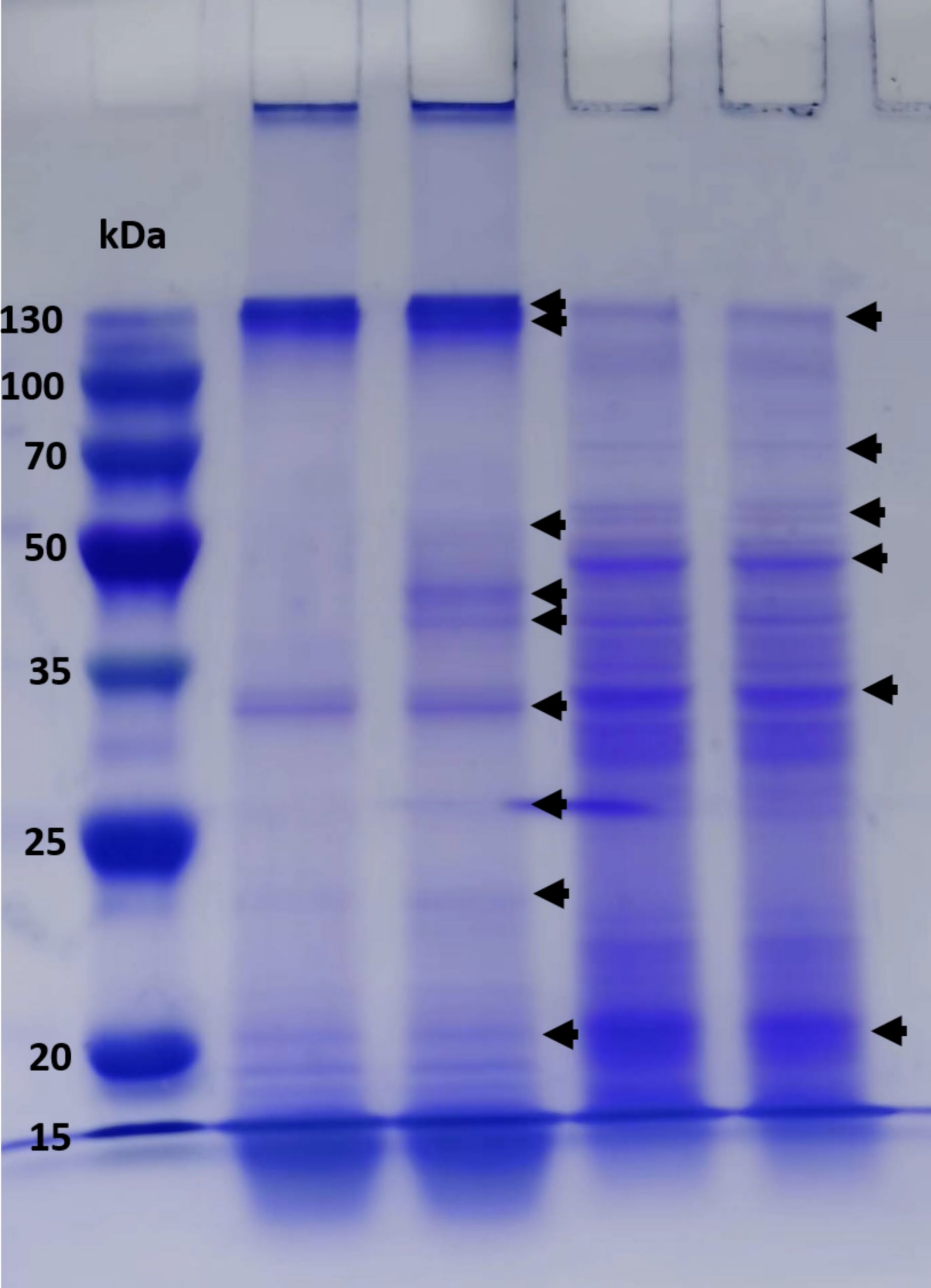
SDS-PAGE of spore-crystal mixture from local *B. thuringiensis* strains. Lane 1: protein molecular mass markers (15 to 130 kDa) (Sangon-Biotech, Shanghai); lane 2 and 3: *B. thuringiensis*-A4 powder formulation and lanes 4 and 5: *B. thuringiensis*-A4 fermentation. Black arrows indicate the different Cry and Cyt proteins. Original SDS-PAGE gel was supplied in the Supplementary Fig. 4

The eight *B. thuringiensis* isolates (A2, A4, A8, A10, B6, C2, C4 and C5) were tested for positive amplification of the *cry* and *cyt* genes using the previously described specific primers. The most frequent genes of the new *B. thuringiensis* isolates analyzed were *cry1*, *cry11Ba*, *cry4Aa*, *cry10Aa*, *cry11Aa*, *cry10*, and *cry11* (Fig. 3A). The isolate with the most genes was *B. thuringiensis* A4, with the amplification of 12 genes (*cry1*, *cry4*, *cry4Aa*, *cry4A*, *cry4B*, *cry10*, *cry10Aa*, *cry11*, *cry11Aa*, *cry11Ba*, *cry32*, and *cyt2* genes) (Fig. 3B). The *B. thuringiensis* A2, B6, and C5 isolates also showed a high number of *cry* and *cyt* genes (Table 3). The *cry2*, *cry4Ba*, *cry24*, *cyt1*, *cyt1Ba*, and *cyt2Aa* genes were not detected in the *B. thuringiensis* isolates evaluated (Table 3). The *cry44Aa* and *cyt2* genes were the least frequent, with each being found in the A2 and A4 isolates, respectively. Herewith, the protein pattern was determined in the *B. thuringiensis* A4 strain in the powder formulation and after fermentation (Supplementary Fig. 2). A range of proteins was observed between 15 and 130 kDa (Fig. 4). Most of these proteins had a high expression level according to the signal detected in the SDS-PAGE analysis. The Cry32, Cry4A, and Cry1 proteins were observed in the range between 129 and 138 kDa, while the Cry4, Cry10, Cry10A, Cry11, Cry11Aa, and Cry11Ba proteins were expressed in the range between 71 and 80 kDa. Additionally, the Cry4B and Cyt2 proteins were located at 54 kDa and 28 kDa, respectively (Fig. 4).

**Table 3:** Detection of *cry* and *cyt* genes in *B. thuringiensis* strains

Genes	*B. thuringiensis* isolates							
	A2	A4	A8	A10	B6	C2	C4	C5
***Cry1***	X	X	X	X	X	X	X	X
***Cry2***								
***Cry4***		X						X
***Cry4Aa***		X	X	X	X	X		
***Cry4Ba***								
***Cry4A***		X						X
***Cry4B***		X						
***Cry10***	X	X				X		X
***Cry10Aa***	X	X			X		X	X
***Cry11***	X	X		X	X			
***Cry11Aa***	X	X			X		X	X
***Cry11Ba***	X	X	X	X	X			X
***Cry24***								
***Cry32***		X			X			
***Cry44Aa***	X							
***Cyt1***								
***Cyt2***		X						
***Cyt1Aa***					X			X
***Cyt1Ba***								
***Cyt2Aa***								

### Mosquito Bioassay using *B. thuringiensis* spores-crystals mixture

**Fig. 5:**
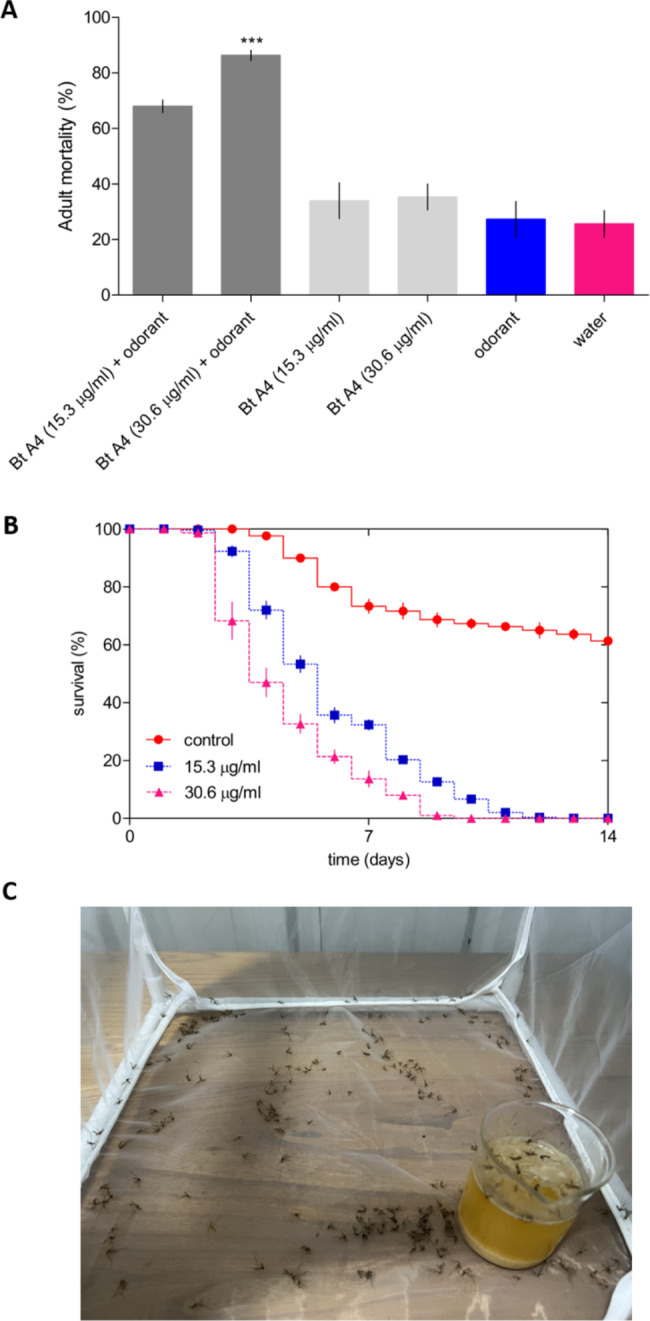
Biological activity of spore-crystal mixture from local strain A4 from *B. thuringiensis* against *C. pipiens pallens* adult. **A**: Effect of combination of natural odorant compounds and spore-crystal mixture from *B. thuringiensis* A4 strain (Bt A4) at different concentrations after seven days post-treatment. **B**: Adult survival using different concentration and time-points. **C**: *C. pipiens pallens* adult treated with combination of natural odorant compounds and *B. thuringiensis* A4 strain (30 µg/ml of total proteins) after 12 days post-treatment. Each bar represents mean values with standard errors of two independent experiments (n = 85 × 2). ***P < 0.05

The screening of the larvicidal bioassays revealed that the *B. thuringiensis* spore-crystal mixture isolates showed promising insect larvicide activities. The LC_50_ and LC_95_ showed a range of lethal concentrations of 1.4 ± 0.5 to 28.5 ± 4.2 µg/ml and 15.3 ± 4.4 to 130.3 ± 14.2 µg/ml, respectively. The native *B. thuringiensis* strain A4 spore-crystal mixture exhibited significantly higher activity against *C. pipiens pallens* larvae (LC_50_: 1.4 ± 0.5 µg/ml and LC_95_: 15.3 ± 4.4 µg/ml; 5.1 × 10^7^ CFU/µg per mg powder) ((**Supplementary Fig. **5), followed by *B. thuringiensis* strains A10, C2, and A2 spore-crystal mixture. The *B. thuringiensis* strain B6 spore-crystal mixture showed the lowest performance, since it had the highest LC_50_ and LC_95_ values of 28.5 ± 4.2 µg/ml and 130.3 ± 14.2 µg/ml, respectively (Table 4). In an independent experiment using the *B. thuringiensis* strain A4 spore-crystal mixture and odorant natural compounds, the adult mortality was evaluated compared to the control conditions. The treatment with the combination of *B. thuringiensis* strains A4 spore-crystal mixture (15 µg/ml of total proteins) and odorant natural compounds displayed significantly high mortality of mosquito adults (68%) compared with the control treatments (Fig. 5A). Meanwhile, a high concentration (30.6 µg/ml of total proteins) produced 86% of adult mortality after 7 days post-treatment (Fig. 5A). The treatment without odorant compounds and *B. thuringiensis* strains A4 spore-crystal mixture alone had not marked influence in the adult mortality (Fig. 5A). There was no adult survival using a higher concentration of the combination (30.6 µg/ml of total proteins) after 10 days of treatment, while the lower concentration (15 µg/ml of total proteins) had the lowest survival after 12 days of treatment (Fig. 5B, C).

**Table 4:** Toxicity of preparations of spores and crystal mixture from eight *B. thuringiensis* isolates against third-instar larval stage of *C. pipiens pallens* after 24 h post-treatment

*B. thuringiensis*-code	LC_50_ (µg/ml) ^a^	LC_95_ (µg/ml) ^a^	CFU/µg × 10^7 b^
**A2**	7.1 ± 1.2	40.7 ± 6.6	5.1 ± 0.3
**A4**	1.4 ± 0.5	15.3 ± 4.4	1.5 ± 0.1
**A8**	16.6 ± 2.7	99.1 ± 12.4	2.3 ± 0.2
**A10**	3.7 ± 1.1	27.1 ± 4.5	1.7 ± 0.1
**B6**	28.5 ± 4.2	130.3 ± 14.2	9.3 ± 0.4
**C2**	6.4 ± 2.7	50.2 ± 3.4	2.3 ± 0.2
**C4**	14.7 ± 3.9	59.4 ± 4.5	1.9 ± 0.3
**C5**	10.2 ± 3.4	84.5 ± 7.7	2.1 ± 0.1

^a^ LC_50_ and LC_95_, Lethal Concentration that kills 50 and 95% of larval population (n = 25), respectively (calculated by Probit analysis and reported with 95% confidence intervals)

^b^ CFU, colony forming unit (mean ± standard error). Each bioassay comprised five concentrations, three replicate for each concentration, and were repeated five times

## Discussion

This work addresses the isolation, identification, characterization, and use of soil microorganisms for vector control, specifically through the use of preparations containing *B. thuringiensis* spores and crystals for the control of mosquito larvae and adults. Although 235 soil samples from three different regions were analyzed, the number of *B. thuringiensis*-related isolates was low. Among the bacterial isolates from the soil, eight isolates were identified as *B. thuringiensis* producing parasporal crystals during sporulation. Molecular analyses of the 16 S rRNA region allowed the identification of *B. thuringiensis* in only eight colonies. On the other hand, through the light and scanning electron microscopes, it was possible to appreciate the different characteristics of the spores and endotoxins produced by *B. thuringiensis*. As for the endotoxins, different forms were observed.

An important aspect of the characterization of *B. thuringiensis* isolates was the determination of the frequency and number of the *cry* and *cyt* genes. This allows an approximation of the possible toxicity of the isolates to the different species of existing mosquitoes. Sometimes, a greater number of *cry* and *cyt* genes in a *B. thuringiensis* isolate is not associated with greater toxicity. However, in other cases, the presence of a greater number of *cry* and *cyt* genes could be related to a greater diversity of production of Cry and Cyt proteins, which can help to avoid insect resistance and increase the spectrum of action against various species of mosquitoes [31, 55–57].

Different profiles of toxic *cry* and *cyt* genes were detected among the evaluated *B. thuringiensis* isolates, and six genes were not detected in the *B. thuringiensis* isolates analyzed. This profile variation might be attributed to the high frequency of genetic information exchange between *B. thuringiensis* bacteria, which allows for variance in genes within a single isolate [44]. These novel bacterial gene combinations can result in isolates with gene compositions unique to distinct kinds of insects, and *B. thuringiensis* effectiveness in insect control may be due to its broad range of activity against diverse groups [14, 58, 59]. Besides, the genetic variety of the novel *B. thuringiensis* isolates in the three ecosystems where these were collected supported the findings of previous research that found genetic variability in Cry and Cyt proteins between samples of isolates collected from different habitats [15, 60, 61].

None of the *B. thuringiensis* isolates harbored all of the investigated *cry* and *cyt* genes, but this result is perhaps expected since none of the known *B. thuringiensis* species or subspecies presents all of the potential genes. However, some isolates may present high toxicity to susceptible insects even with a synthetic composition of *cry*-*cyt* genes [62]. The number of genes in a single isolate does not appear to be the factor determining the immediate death of the susceptible insect per se, but it may affect the toxic activity of bacteria when they encounter optimal conditions in the gut of the insect.

The δ-endotoxins are divided into two protein families, Cry and Cyt, which are often generated by *B. thuringiensis* subspecies. The presence of Cry and Cyt proteins in the same subspecies is common in Diptera-targeting strains. The Cry family of proteins comprise mainly 74 distinct kinds that are encoded by 770 different *cry* genes, whereas the Cyt family is of three different types that are encoded by 38 different *cyt* genes [63]. The specificity of the number of toxic genes in each isolate, as well as the vulnerability of the larvae and the very alkaline pH of the gut, encourage the solubilization and release of the protein crystals, all of which are essential determinants determining the efficiency of the bacterial toxicity against larvae [10, 64].

The number of genes in *B. thuringiensis* isolates can lack the correlation between the number of amplified genes and the intensity of the toxic activity in the mosquito gut [60, 65, 66]. Among the twenty genes evaluated in this study, including fifteen in the Cry family and five in the cyt family, the most frequent were the *cry1*, *cry11Ba*, *cry4Aa*, *cry10Aa*, *cry11Aa*, *cry10*, and *cry11* genes of the new *B. thuringiensis* isolates. The *cry1* and *cry10* genes were more frequent in the *B. thuringiensis* isolates previously described [55, 66–68]. The combination of the Cry4, Cry10, Cry11, and Cyt toxins are known to have a potent activity against mosquitoes [69]. Combinations of Cry4Aa with Cry11Ba proteins have been found to interact synergistically with a significant high toxicity against mosquito species *Culex pipiens* [70]. Interesting, this combination was detected in the *B. thuringiensis* A4, A8, A10 and B6 strains, when the *cry4Aa* with *cry11Ba* genes were analyzed.

Preparation of spore-crystal mixture from local *B. thuringiensis* isolates exhibited larvicidal activity. Several preparations of spore-crystal mixture from *B. thuringiensis* strains with good mosquitocidal activity have been isolated from various parts of the world [19, 62, 71, 72]. Among the preparation of spore-crystal mixture from *B. thuringiensis* isolates with significantly enhanced larvicidal activity identified in this study, the preparation with isolate A4 was the most toxic with bioactivity against *C. pipiens pallens* larvae (LC_50_: 1.4 ± 0.5 µg/ml and LC_95_: 15.3 ± 4.4 µg/ml; 5.1 × 10^7^ CFU/µg per mg powder). The bioactivity of preparation from isolate A4 showed an interesting low LC_50_ and LC_95_ concentration, compared with previous works where the concentrations of preparation of the new *B. thuringiensis* isolates were higher, including the reference *B. thuringiensis* subsp. *israelensis* H14 strain [2]. However, preparations with the reference strain and new *B. thuringiensis* isolates had a lower LC_50_ concentration against *Culex pipiens* [68] or *Aedes aegypti* larvae, respectively [73]. The comparison is a suitable approximation of the effectiveness of preparation of spore-crystal mixture from isolated *B. thuringiensis*, but this should not be taken as a final criterion, without a real-time comparison with reference isolates or similar isolates. Although, the behavior of the reference isolates may vary in relation to the mosquito species and the experimental conditions of the region. This is the reason for the continuous search for native *B. thuringiensis* strains to be used in the specific regions where they were previously isolated.

The use of *B. thuringiensis* as a mosquito larvicide is widely known, previous research on its biological effects on adult mosquitos has shown interesting and contradictory results [36, 37, 40, 74–76]. This study determined that the mixture of *B. thuringiensis* and natural odorant caused significant mortality of mosquito adults after ingestion. These odorant baits are one alternative strategy to attract mosquitoes. Also, the probable presence of attractive toxic sugar in the natural compounds can have an additive effect on mortality. Both attractive toxic sugar baits and attractant compound present in fruits have been shown to be effective against several important mosquito vectors [77, 78]. The results show an interesting alternative to mosquito adult control. However, further studies are needed in the field conditions using *B. thuringiensis* and natural odorants on wild mosquito species populations.

The new preparations of spore-crystal mixture of *B. thuringiensis* isolates from Rizhao soil are promising for the biological control of *C. pipiens pallens* larvae, which can be evidenced by the biological activity in larvae caused by the Cry and Cyt toxins, making these isolates highly toxic and enabling them to be safely used as a biotechnological tool for the manufacture of biological larvicide with different combinations of toxins from those currently used. We believe that the discovery of the A4 strain may prove crucial for future bioinsecticide production against mosquito vectors. This is due to its greatly enhanced mosquitocidal activity against *C. pipiens pallens*, combined with its possible economic and environmental advantages. In the trials, a comparison with *B. thuringiensis* subsp. *israelensis* isolates would have been useful. The objective was to isolate and compare novel native isolates under laboratory conditions. However, these results only reach a candidate product. Assays on a small, medium, and large scale will be required in natural conditions using a final product formulated. In the future, a comparison of the final product formulated with other commercial pesticides would be required.

## Electronic supplementary material

Below is the link to the electronic supplementary material.

**Supplementary Figure 1**. Map of Rizhao showing the locations of the sites from which the soil samples were collected (stars). Stars indicate sites from which toxic *Bacillus thuringiensis* was isolated. **Supplementary Figure 2**. Schematic representation of spore-crystal mixture from *B. thuringiensis* and natural odorant production to the control of larvae and adult mosquitoes. **Supplementary Figure 3**. Schematic representation of the evaluation of biological activity preparation of spore-crystal mixture from *B. thuringiensis* against larvae (**A**) and adult (**B**) mosquitoes under controlled conditions. **Supplementary Figure 4**. Original full-length SDS-PAGE gels of spore-crystal mixture from local *B. thuringiensis* strains. **Supplementary Figure 5**. Evaluation of LC50 of spore-crystal mixture from *B. thuringiensis* strain A4 using Probit, arithmetic, and logarithmic analyses. Strain A4 and one replication.

## Data Availability

All data generated or analyzed during this study are included in this published article and its supplementary information files.
