# Supplementary material for: New native Bacillus thuringiensis strains induce high insecticidal action against Culex pipiens pallens larvae and adults
Source: BMC Microbiol. 2023 Apr 13;23:100. doi: 10.1186/s12866-023-02842-9 (PMC10099900; doi:10.1186/s12866-023-02842-9)
Supplement: Supplementary file 1 — Supplementary Figure 1. Map of Rizhao showing the locations of the sites from which the soil samples were collected (stars). Stars indicate sites from which toxic Bacillus thuringiensis was isolated. Supplementary Figure 2. Schematic representation of spore-crystal mixture from B. thuringiensis and natural odorant production to the control of larvae and adult mosquitoes. Supplementary Figure 3. Schematic representation of the evaluation of biological activity preparation of spore-crystal mixture from B. thuringiensis against larvae (A) and adult (B) mosquitoes under controlled conditions. Supplementary Figure 4. Original full-length SDS-PAGE gels of spore-crystal mixture from local B. thuringiensis strains. Supplementary Figure 5. Evaluation of LC50 of spore-crystal mixture from B. thuringiensis strain A4 using Probit, arithmetic, and logarithmic analyses. Strain A4 and one replication. [file 12866_2023_2842_MOESM1_ESM.pdf]

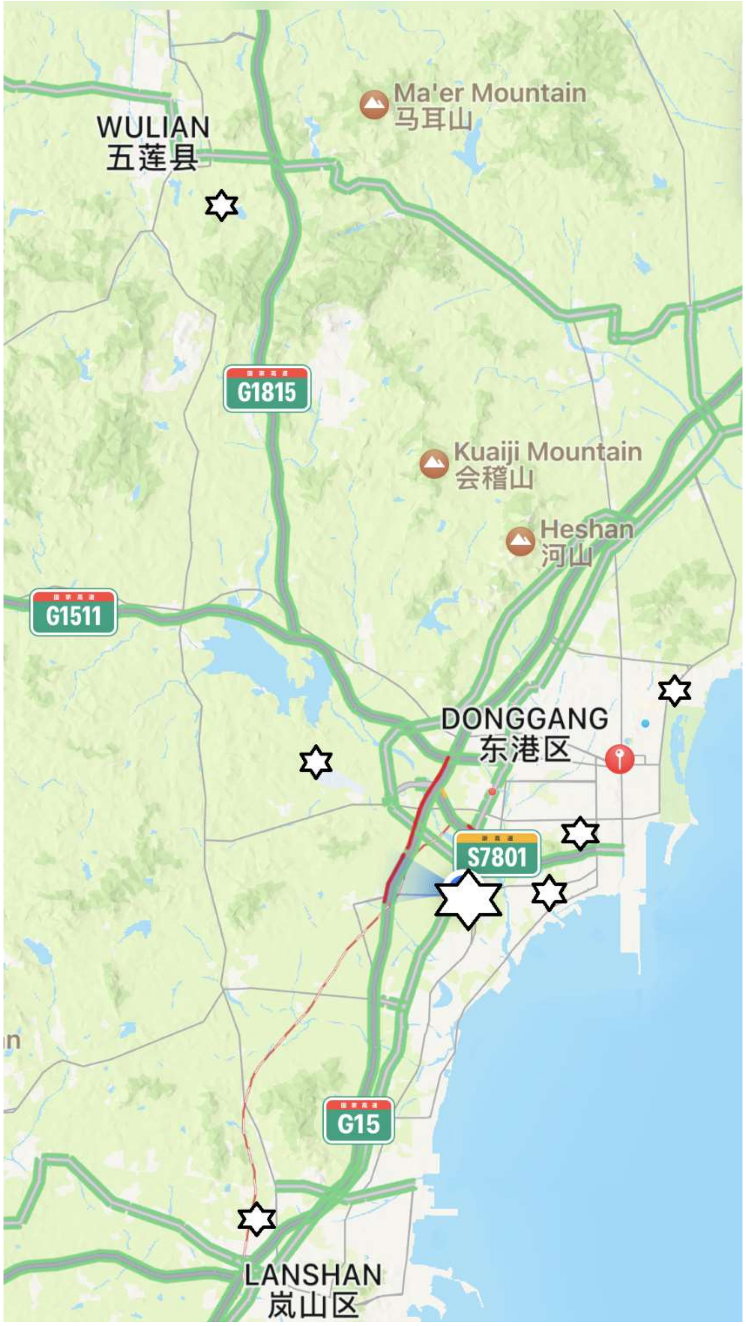

Supplementary Figure 1. Map of Rizhao showing the locations of the sites from which the soil samples were collected (stars). Stars indicate sites from which toxic *Bacillus thuringiensis* was isolated

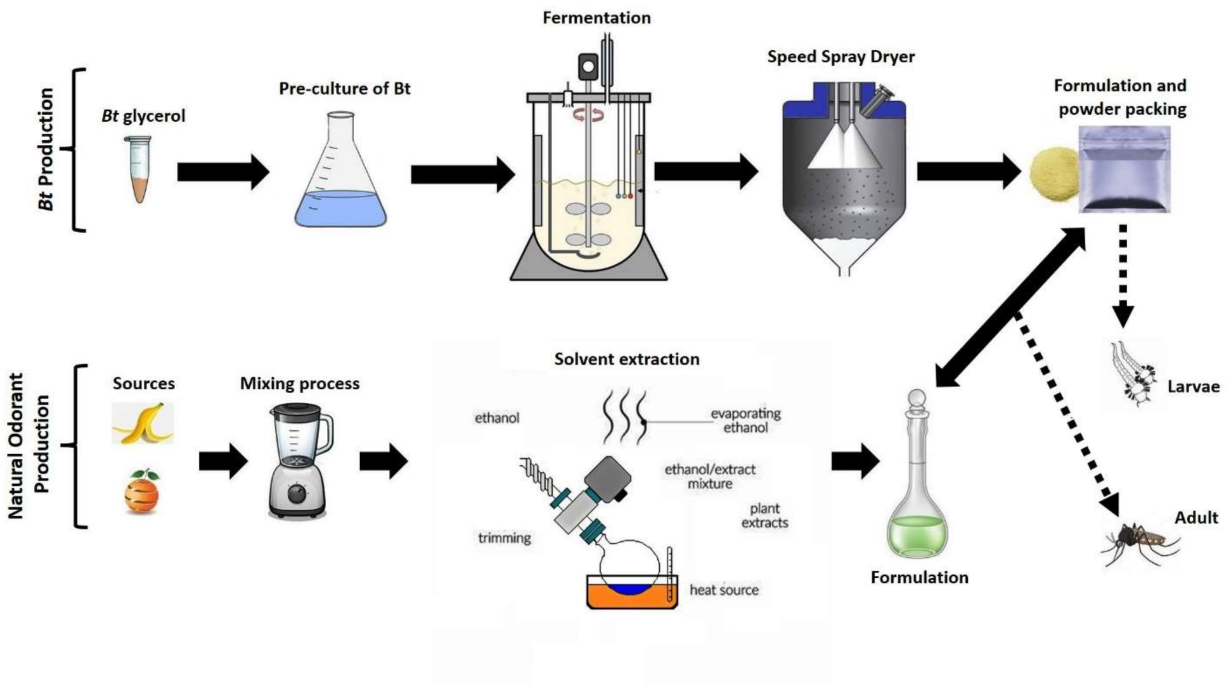

Supplementary Figure 2. Schematic representation of spore-crystal mixture from *B. thuringiensis* and natural odorant production to the control of larvae and adult mosquitoes

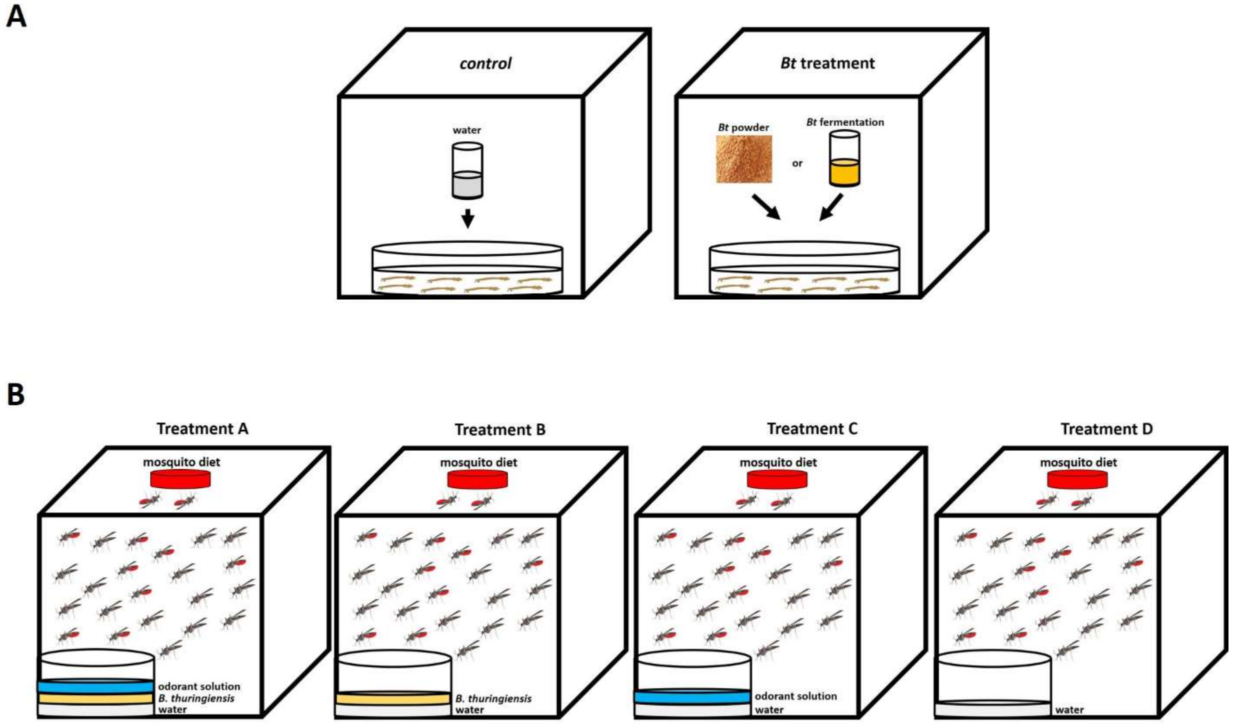

Supplementary Figure 3. Schematic representation of the evaluation of biological activity preparation of spore-crystal mixture from *B. thuringiensis* against larvae (**A**) and adult (**B**) mosquitoes under controlled conditions

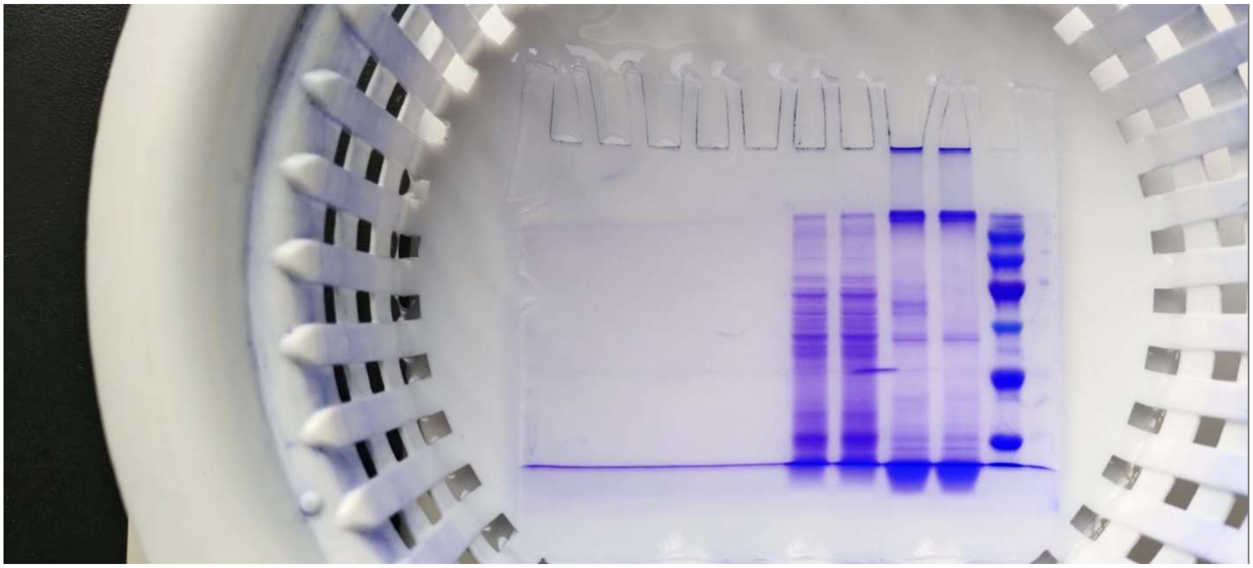

Supplementary Figure 4. Original full-length SDS-PAGE gels of spore-crystal mixture from local *B. thuringiensis* strains.

Supplementary Figure 5. Evaluation of LC_{50} of spore-crystal mixture from *B. thuringiensis* strain A4 using Probit, arithmetic, and logarithmic analyses. Strain A4 and one replication.

		**Dead Larvae**	**Arithmetical analysis**	**Logarithmic analysis**	**Logarithmic concentration**	**Probit analysis**
**Concentration (µg/ml)**	**Number of Larvae**	**After 24 hours**	**After 24 hours**	**After 24 hours**		**After 24 hours**
7	25	24	96	4	0.84509804	6.750686071
6	25	24	96	4	0.77815125	6.750686071
5	25	24	96	4	0.698970004	6.750686071
4	25	22	88	12	0.602059991	6.174986792
3	25	19	76	24	0.477121255	5.706302563
2	25	17	68	32	0.301029996	5.467698799
1	25	16	64	36	0	
0	25	0	0	100	0	
**y=mx+b**			**Arithmetical analysis**		**Logarithmic analysis**	**Probit analysis**
**m**			11.048		-80.422	2.7419
**b**			34.333		64.22	4.5749
**y**			50		50	5
**x=(y-b)/m**		**LC_{50}**	**1.418084721**		0.176817289	0.155038477
				**LC_{50}**	**1.502509715**	**1.429020559**

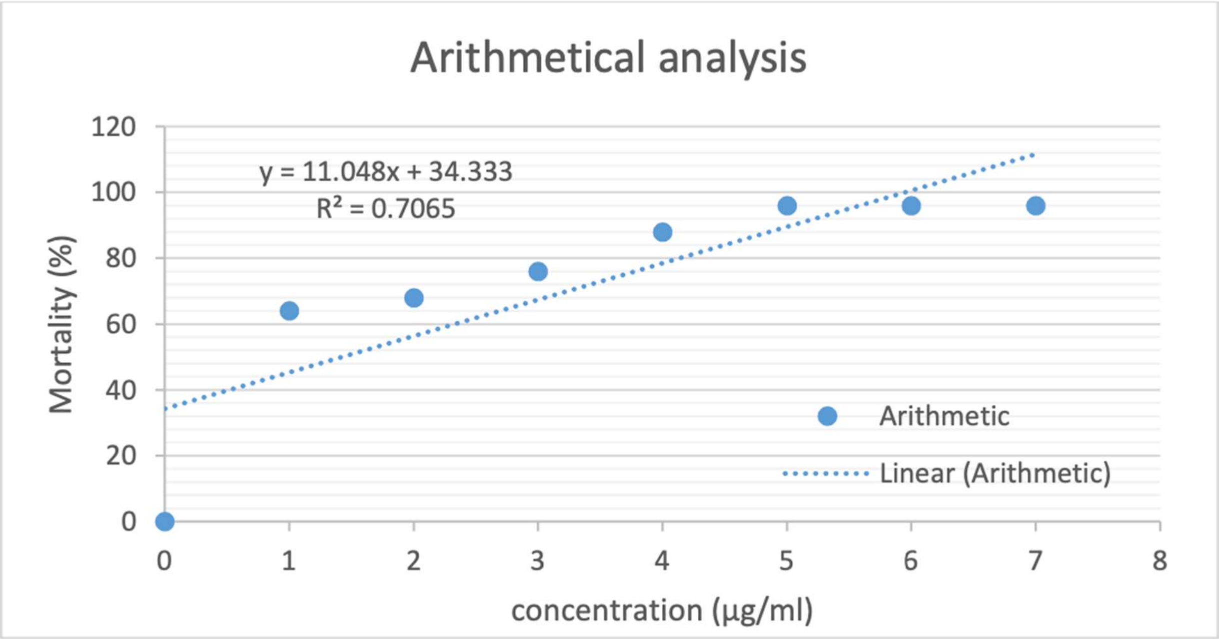

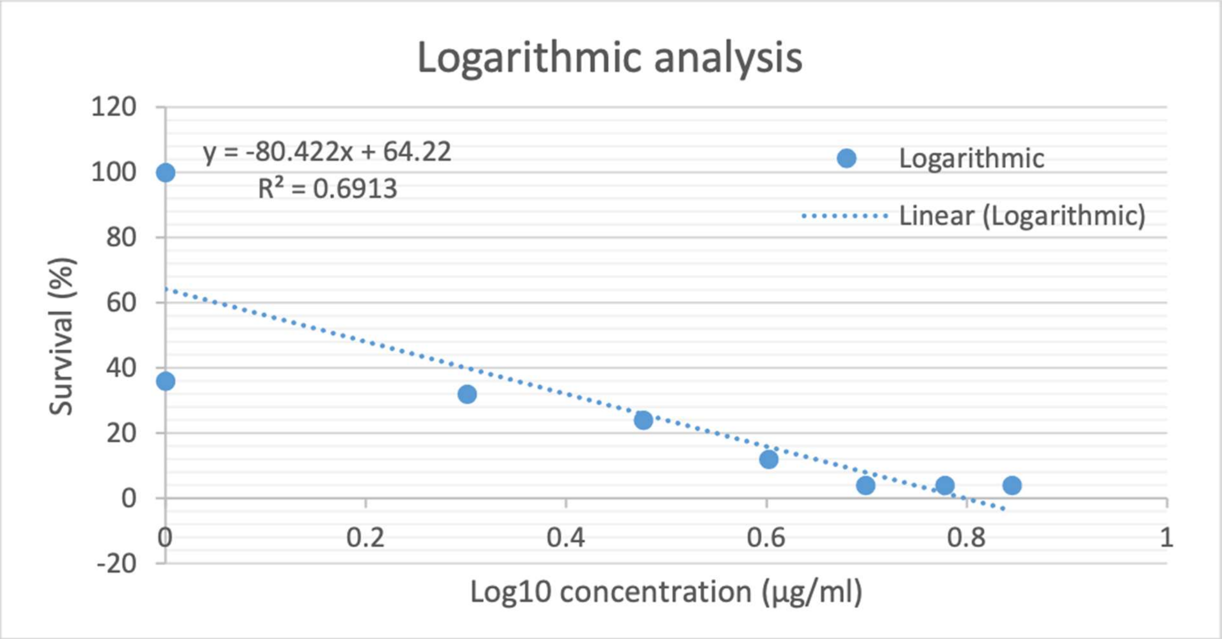

Logarithmic analysis
y = -80.422x + 64.22
R² = 0.6913
Logarithmic
Linear (Logarithmic)
Survival (%)
120
100
80
60
40
20
0
-20
0
0.2
0.4
0.6
0.8
1
Log10 concentration (µg/ml)

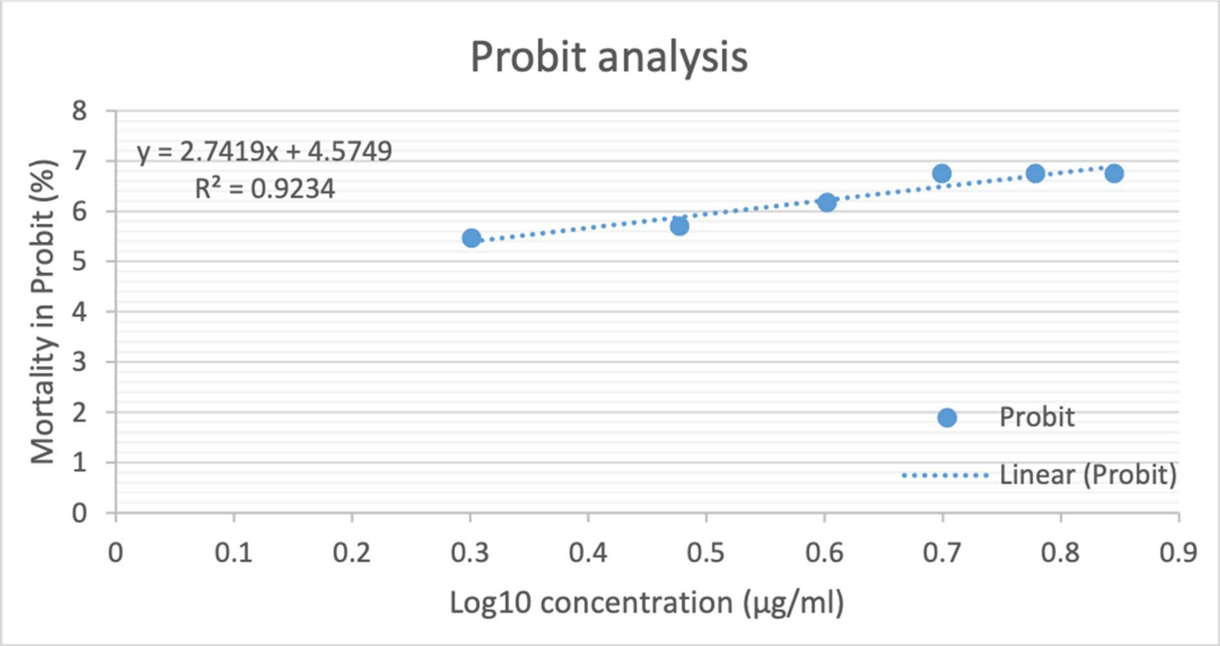

Probit analysis
y = 2.7419x + 4.5749
R² = 0.9234
Probit
Linear (Probit)
Mortality in Probit (%)
8
7
6
5
4
3
2
1
0
0
0.1
0.2
0.3
0.4
0.5
0.6
0.7
0.8
0.9
Log10 concentration (µg/ml)
